# Supplementary material for: Clusters of cause specific neonatal mortality and its association with per capita gross domestic product: A structured spatial analytical approach
Source: PLoS One. 2021 Aug 17;16(8):e0255882. doi: 10.1371/journal.pone.0255882 (PMC8370610; doi:10.1371/journal.pone.0255882)
Supplement: Attachment — Submitted filename: Response to Reviewers.docx [file pone.0255882.s001.docx]

**PONE-D-20-35565
Clusters of cause specific neonatal mortality and its association with per capita gross domestic product: a structured spatial analytical approach
PLOS ONE**

We would like to thank the reviewers for their thoughtful suggestions. The responses to the Editor and reviewers are below:

**Editor**

1. Please ensure that your manuscript meets PLOS ONE's style requirements, including those for file naming.

 We checked the PLOS ONE´s style requirements and follow them.

2. In the ethics statement in the manuscript and in the online submission form, please provide additional information about the patient records/samples used in your retrospective study, including: a) whether all data were fully anonymized before you accessed them; b) the date range (month and year) during which the data were accessed.

We added a sentence to the manuscript to include the information requested: “The dataset was accessed from October 2018 to June 2021, and it was fully anonymized before the access.

3. Thank you for stating the following in the Funding Section of your manuscript:

[Fundação de Amparo à Pesquisa de São Paulo-FAPESP, Project # 2017/03748-7]

We note that you have provided funding information that is not currently declared in your Funding Statement. However, funding information should not appear in the Acknowledgments section or other areas of your manuscript. We will only publish funding information present in the Funding Statement section of the online submission form.

Please remove any funding-related text from the manuscript and let us know how you would like to update your Funding Statement. Currently, your Funding Statement reads as follows:

 [The datasets generated during and/or analysed during the current study are available from the corresponding author on reasonable request.]

 Please include your amended statements within your cover letter; we will change the online submission form on your behalf.

We would like to update our Funding Statement as: Fundação de Amparo à Pesquisa de São Paulo - FAPESP, Project # 2017/03748-7.

4. We note that you have indicated that data from this study are available upon request. PLOS only allows data to be available upon request if there are legal or ethical restrictions on sharing data publicly. For information on unacceptable data access restrictions, please see http://journals.plos.org/plosone/s/data-availability#loc-unacceptable-data-access-restrictions.

 In your revised cover letter, please address the following prompts:

 a) If there are ethical or legal restrictions on sharing a de-identified data set, please explain them in detail (e.g., data contain potentially identifying or sensitive patient information) and who has imposed them (e.g., an ethics committee). Please also provide contact information for a data access committee, ethics committee, or other institutional body to which data requests may be sent.

 b) If there are no restrictions, please upload the minimal anonymized data set necessary to replicate your study findings as either Supporting Information files or to a stable, public repository and provide us with the relevant URLs, DOIs, or accession numbers. Please see http://www.bmj.com/content/340/bmj.c181.long for guidelines on how to de-identify and prepare clinical data for publication. For a list of acceptable repositories, please see http://journals.plos.org/plosone/s/data-availability#loc-recommended-repositories.

 We will update your Data Availability statement on your behalf to reflect the information you provide.

We uploaded the database of the study in the ZENODO public repository as requested: [https://zenodo.org/deposit/4915807](about:blank). The DOI for the data is: 10.5281/zenodo.4915807. The dataset is in closed access right now, but we will change to public access if the paper is accepted. Please update the Data Availability statement on our behalf.

5. We note that Figures 2, 3 and 4 in your submission contain map images which may be copyrighted. All PLOS content is published under the Creative Commons Attribution License (CC BY 4.0), which means that the manuscript, images, and Supporting Information files will be freely available online, and any third party is permitted to access, download, copy, distribute, and use these materials in any way, even commercially, with proper attribution. For these reasons, we cannot publish previously copyrighted maps or satellite images created using proprietary data, such as Google software (Google Maps, Street View, and Earth). For more information, see our copyright guidelines: http://journals.plos.org/plosone/s/licenses-and-copyright.

 We require you to either (1) present written permission from the copyright holder to publish these figures specifically under the CC BY 4.0 license, or (2) remove the figures from your submission:

 You may seek permission from the original copyright holder of Figures 2, 3 and 4 to publish the content specifically under the CC BY 4.0 license.

 We recommend that you contact the original copyright holder with the Content Permission Form ([http://journals.plos.org/plosone/s/file?id=7c09/content-permission-form.pdf](about:blank)) and the following text:

“I request permission for the open-access journal PLOS ONE to publish XXX under the Creative Commons Attribution License (CCAL) CC BY 4.0 ([http://creativecommons.org/licenses/by/4.0/](about:blank)). Please be aware that this license allows unrestricted use and distribution, even commercially, by third parties. Please reply and provide explicit written permission to publish XXX under a CC BY license and complete the attached form.”

 Please upload the completed Content Permission Form or other proof of granted permissions as an "Other" file with your submission.

 In the figure caption of the copyrighted figure, please include the following text: “Reprinted from [ref] under a CC BY license, with permission from [name of publisher], original copyright [original copyright year].”

 If you are unable to obtain permission from the original copyright holder to publish these figures under the CC BY 4.0 license or if the copyright holder’s requirements are incompatible with the CC BY 4.0 license, please either i) remove the figure or ii) supply a replacement figure that complies with the CC BY 4.0 license. Please check copyright information on all replacement figures and update the figure caption with source information. If applicable, please specify in the figure caption text when a figure is similar but not identical to the original image and is therefore for illustrative purposes only.

The maps in figures 2, 3 and 4 are not copyrighted. They were created by the research team and all the authors give permission for the open-access journal PLOS ONE to publish them under the Creative Commons Attribution License (CCAL) CC BY 4.0.

**Reviewer #1**

The manuscript provides very useful insight into the cause specific neonatal mortality and its association with per capita gross domestic product in upper middle-income settings. The methodology of the study is sound and intelligent inferences are made from the statistical analysis. There are a few minor suggestions
Introduction: The Introduction can be further strengthened by clearly setting out the aim of the study highlighting the significance of strengthening the evidence base in this area and why this study is important.

Thank you for your suggestion. We added a sentence at the end of the introduction (before the aim of the study) summarizing the importance of the study: “Since asphyxia/hypoxia is a leading cause of pottentialy preventable neonatal deaths in Brazil, a methodology to identify high risk areas for these deaths and to explore the co-occurrence of associated events would be helpful for guiding public health strategies.”

**Reviewer #2**

The authors conducted a population based study to investigate the cluster areas of asphyxia-associated neonatal mortality and to explore the per capita gross domestic product (GDP) as an associated risk factor in São Paulo State, Brazil. Ecological study including live births residents in SP from 2004-2013. They concluded that Spatial analysis identified cluster areas of high asphyxia-associated neonatal mortality and low GDP rates, with a significant negative correlation. Overall, the manuscript was well written. I have some comments on the methodology and analysis.

1. One of the major limitations of the study is that there is no adjustment on the other risk factors such as education, population density, income, etc. into their analysis. Is that due to the lack of the information?

Several other factors may impact the distribution of asphyxia-associated neonatal mortality in São Paulo State as stated by the reviewer: education, income, medical training, health care facility distribution, quality of prenatal care, among others. The aim of our study was not to analyze risk factors for asphyxia-associated neonatal deaths, but to identify the cluster areas where these deaths occur and to explore if the methodology of spatial analysis is helpful to identify risk factors associated to the geographic distribution of asphyxia-associated neonatal death. In this context, we described the aim of our study as “to investigate by a structured and hierarchical spatial analytical method the occurrence of clustering areas of asphyxia-associated neonatal mortality and ***to explore*** the mean per capita GDP as an associated risk factor…” Future studies need to be done in order to identify additional risk factors. We reinforced this idea at the end of the discussion section adding the sentence: “Certainly other risk factors are associated with asphyxia-associated neonatal mortality and should be studied. The correlation between neonatal asphyxia-associated deaths and per capita GDP was exploratory and it was not adjusted for other risk factors.”

2. The authors need to provide a summary table for the demographic factors for the study cohort.
We included the demographic characteristics for the study cohort on the first paragraph of the results section: “The mean birth weight of the neonates that died with asphyxia was 1,907g (±SD 1,154g); with 17 infants with unknown birth weight. More than half were male (56% [3752/6713]. Regarding gestational age, 37% [2,484/6,642] had 37 to 41 weeks, 15% [978/6,642] 32 to 36 weeks, 16% [1,036/6,642] 28 to 31 weeks, 32% [2,090/6,642] 22 to 27 weeks, and 1% [54/6,642] ≥42 weeks, with 71 infants with unknown gestational age. Maternal age was <20 years for 21% [1,391/6,713], 20-34 years for 65% [4,361/6,713], and > 34 years for 14% [961/6,713].”

3. The authors conducted spatial moving average maps showed a non-random distribution among municipalities, with presence of clusters (I=0.049; p=0.026). They also conducted local Bayes estimates, and identified much more significant clusters (I=0.587; p=0.001). Why there is big difference between these the results of these two methods?
The difference found shows the importance of the methodology. Before the smoothing approach, the outliers (areas with small populations) diluted the effect of the clusters. After using the local Bayes estimates to correct for random spatial fluctuation, we could identify the clusters areas more precisely. The previous sentence was included in the discussion section.

4. On Page 15, more detailed explanation needs to be provided for the formulae on line 171.

We added more information explaining the formulae for the local empiric Bayesian estimator equation: “The larger the population in area *I*, the closer weight *wi*, which means that in areas where the population is larger, the empirical Bayes estimate is very close to the risk rate for that area (*ti*), and in areas with a small population, the value of *bi* will be intermediary between *ti* and *mi.”*

5. What is the significance level of the study? It should be described in the method section.

“A p-value <0.05 was considered as statistically significant for all analyses.” We added this sentence at the end of the methods section.

6. I didn’t find ethnical approval for this study.

Thank you for your note. We added the ethical approval for the study after the first paragraph in the methods section: “The study was approved by the Ethics Committee on Human Research of Escola Paulista de Medicina – Universidade Federal de São Paulo (#2,580,929) and by the Board of Directors of Fundação SEADE”.

**Reviewer #3**

 The study focuses on an interesting and needed topic and the analysis is sounds.
However, there are some relevant limitations. The main one is linked to the statement presented in lines 378-380 [“Therefore, the proposed methodology may be helpful for guiding public health efforts to decrease avoidable causes of neonatal mortality”]. This statement is not fully truth. It is of course extremely useful to assess the distribution of neonatal mortality but the usability of the link with GPD will require further analysis. In fact, other factors could count for this link, as stated by the authors themselves, like health service quality and availability/accessibility and demographic factors (mother age and/or education, for example). None of these data have been considered in order to actually find the factor linked with higher mortality, nor these have been considered in order to adjust the GDP as risk factors. Practically speaking the found relationship is not actually useful to defined which actions to consider reducing neonatal mortality in these areas.

Several other factors may impact the distribution of asphyxia-associated neonatal mortality in São Paulo State as stated by the reviewer: education, income, medical training, health care facility distribution, quality of prenatal care, among others. The aim of our study was not to analyze risk factors for asphyxia-associated neonatal deaths, but to identify the cluster areas where these deaths occur and to explore if the methodology of spatial analysis is helpful to identify risk factors associated to the geographic distribution of asphyxia-associated neonatal deaths. Future studies need to be done in order to identify the complex interaction of multiple risk factors associated with neonatal deaths in the clusters identified in this study. We reinforce the limitation of the association found between asphyxia-associated mortality and per capita GDP adding the following sentence at the end of the results section: “Certainly other risk factors are associated with asphyxia-associated neonatal mortality and should be studied. The correlation between neonatal asphyxia-associated deaths and per capita GDP was exploratory and it was not adjusted for other risk factors.”

Other key points are the following:
1. The rational for which the study focuses just on asphyxia related neonatal mortality in never stated and unclear. A reason could be that a previous study assessed that link but if that is the case it would be advantageous to clarify this since the beginning instead of just in the discussion. Also, considering the discrepancies between the two studies and the different period it seems even more relevant to explain why avoiding including overall neonatal mortality and possibly, other cause of the same.

We focused our study on the asphyxia-associated neonatal mortality because asphyxia/hypoxia is a leading cause of potentially preventable neonatal deaths in Brazil. We added a sentence in the introduction to highlight the importance of our study: “Since asphyxia/hypoxia is a leading cause of pottentialy preventable neonatal deaths in Brazil, a methodology to identify high risk areas for these deaths and to explore the co-occurrence of associated events would be helpful for guiding public health strategies.” Also, several other studies describe the overall neonatal mortality in Brazil, but there are few, if any, reporting cause-specific neonatal mortality.

2. Please consider adding the proportion of the main causes of neonatal mortality in Brazil (line 79-81)

We added that information as suggested: “Despite its considerable decline over the years, neonatal mortality remains high, with almost 10% of the underlying cause of deaths being classified as asphyxia/hypoxia. Asphyxia/hypoxia is the 5^th^ cause of neonatal mortality in Brazil following prematurity (30%), congenital malformation (23%), infections (19%) and maternal factors, but it represents a pottentialy preventable one”

3. Please consider adding some considerations/hypothesis on why the increase in GDP has only be associated with reduction on post-neonatal mortality in Brazil (line 90-93)

We added the hypothesis given by the authors of the cited study: “For Volpe et al, the increase in public expenditure on health reduced the post-neonatal mortality with no impact on neonatal mortality because the strategies for reducing neonatal mortality are more expensive than those for reducing post-neonatal mortality, for which low-cost public interventions may have a great effect”.

4. Line 127-128: “The mean per capita GDP for the period was calculated for each municipality by dividing the GDP by the estimated population in the same period.” I think it would be clearer adding the bold part “by dividing the GDP of each municipality by the estimated population”, otherwise it looks like that the total SP GDP was divided by people which would imply considering that every person in each municipality has the same level of wealth.

Thanks for the suggestion. We changed the sentence to: “The mean per capita GDP for the period was calculated for each municipality by dividing the GDP of each municipality by the estimated population in the same period.”

5. Result: please add the total neonatal deaths and the distribution per cause (line 188). Also, data on the number of municipalities with high asphyxia related neonatal deaths rate and no association with GDP [paragraph from line 254] should eb added.

The total number of neonatal deaths was 50,993. We added this information to **Figure 1**. Unfortunately, we cannot add the distribution per cause because we just have the database for deaths associated with asphyxia. The study dataset will be available in the ZENODO public respiratory: [https://zenodo.org/deposit/4915807](about:blank).

There were 63 municipalities with high asphyxia-associated neonatal deaths and no significance (by LISA) for mean per capita GDP. We added a sentence with this information at the end of the results section: “There were 63 municipalities in clusters of high asphyxia-associated neonatal deaths but no significance (by LISA) for mean per capita GDP rates”.
