# Supplementary material for: Clusters of cause specific neonatal mortality and its association with per capita gross domestic product: A structured spatial analytical approach
Source: PLoS One. 2021 Aug 17;16(8):e0255882. doi: 10.1371/journal.pone.0255882 (PMC8370610; doi:10.1371/journal.pone.0255882)
Supplement: Attachment — Submitted filename: Response for Reviewers_13jul2021.docx [file pone.0255882.s002.docx]

Response for Reviewer #1 and 2

Thank you for your inputs in the previous revised manuscript.

Response for Reviewer #3:

*Thank you for the replies to the previous questions and the edits to the paper.
However, it is clear even from the authors replies that what has been explored is the association between one specific cause of death and GDP. This association is defined as a “risk factor” but this has not be proven. Only the investigation of others listed risk factors could have clarified whether the risk factor is the low GDP, or, for example, the low access to care, low education, etc. It is sure that low GDP is likely to be associated with such risk factors but “having and association” and “being a risk factors” are two different issues. The authors themselves stated that “The aim of our study was not to analyze risk factors for asphyxia-associated neonatal, but to identify the cluster areas where these deaths occur and to explore if the methodology of spatial analysis is helpful to identify risk factors associated to the geographic distribution of asphyxia-associated neonatal death". Therefore, the manuscript could be considered if the objective is focus in the geospatial distribution but avoiding the definition of GDP as a risk factor (from objective and discussion).*

We completely agree with you. We cannot say that we have studied a risk factor for asphyxia-associated neonatal mortality given the methodology applied. Our objective was to investigate by a spatial analytical approach the occurrence of clustering areas of asphyxia-associated neonatal deaths and to explore the mean per capita GDP distribution in these areas. You are right that we should be more careful with our language in order to avoid misunderstandings. We have reviewed the manuscript to exclude or modify all the sentences that could give the idea that we were studying a risk factor instead of exploring a possible association between per capita GDP and asphyxia-associated neonatal deaths:

- In the objective of the abstract, we changed the following sentence “*To investigate the cluster areas of asphyxia-associated neonatal mortality and to explore the per capita gross domestic product (GDP) as an associated risk factor in São Paulo State (SP), Brazil*” to “To investigate the cluster areas of asphyxia-associated neonatal mortality and to explore its association with per capita gross domestic product (GDP)” (Revised manuscript with tracked changes – lines 45-47).
- In the last sentence of the 4^th^ paragraph of the Introduction section, we changed the sentence from “*Spatial analysis may be used to explore the co-occurrence of a health event and other population events on the same geographic unit (i.e. economic, educational, sociological indicators) as possible risk factors*” to “Spatial analysis may be used to explore the co-occurrence of a health event and other population events on the same geographic unit (i.e. economic, educational, sociological indicators)” (Revised manuscript with tracked changes – lines 104-106).
- We changed the objective at the end of introduction section from “*our aim was to investigate by a structured and hierarchical spatial analytical method the occurrence of clustering areas of asphyxia-associated neonatal mortality and to explore the mean per capita GDP as an associated risk factor*” to “our aim was to investigate by a structured and hierarchical spatial analytical method the occurrence of clustering areas of asphyxia-associated neonatal mortality and to explore its association with mean per capita GDP” (Revised manuscript with tracked changes – lines 110-112).
- In the 8^th^ paragraph of the discussion section, we changed the following: “*Certainly, other risk factors are associated with asphyxia-associated neonatal mortality and should be studied. The correlation between neonatal asphyxia-associated deaths and per capita GDP was exploratory and it was not adjusted for other risk factors*”; to “Certainly, other variables are associated with asphyxia-associated neonatal mortality and should be studied. The correlation between neonatal asphyxia-associated deaths and per capita GDP was exploratory and it was not adjusted for other confounders” (Revised manuscript with tracked changes – lines 392-395).
- In the last paragraph of the discussion section, we changed “*The present optimized, structured, and hierarchical approach allowed for the identification of high-risk areas of asphyxia-associated mortality and an associated risk factor (GDP*)” to “The present optimized, structured, and hierarchical approach allowed for the identification of high-risk areas of asphyxia-associated mortality.” (Revised manuscript with tracked changes – lines 410-411)

We thank you for your thoughtful suggestions to improve our manuscript.
